# Effect of Chitosan Nanoparticle-Loaded *Thymus serpyllum* on Hydrogen Peroxide-Induced Bone Marrow Stromal Cell Damage

**DOI:** 10.1155/2019/5142518

**Published:** 2019-02-26

**Authors:** Salma Baig, Ainnul Hamidah Syahadah Azizan, Hanumantha Rao Balaji Raghavendran, Elango Natarajan, Sangeetha Naveen, Malliga Raman Murali, Hui Yin Nam, Tunku Kamarul

**Affiliations:** ^1^Tissue Engineering Group (TEG), National Orthopaedic Centre of Excellence in Research and Learning (NOCERAL), Department of Orthopaedic Surgery, Faculty of Medicine, University of Malaya, Lembah Pantai, 50603 Kuala Lumpur, Malaysia; ^2^Research Centre for Crystalline Materials, School of Science and Technology, Sunway University, 47500 Selangor, Malaysia; ^3^Department of Chemistry, Faculty of Science, University of Malaya, 50603 Kuala Lumpur, Malaysia; ^4^Faculty of Engineering, UCSI University, Kuala Lumpur, Malaysia

## Abstract

We have determined the protective effects of *Thymus serpyllum* (TS) extract and nanoparticle-loaded TS on hydrogen peroxide-induced cell death of mesenchymal stromal cells (MSCs) *in vitro*. Gas chromatography–mass spectroscopy confirmed the spectrum of active components in the extract. Out of the three different extracts, the hexane extract showed significant free radical scavenging activity. Treatment of MSCs with H_2_O_2_ (hydrogen peroxide) significantly increased intracellular cell death; however, pretreatment with TS extract and nanoparticle-loaded TS (200 *μ*g/ml) suppressed H_2_O_2_-induced elevation of Cyt-c and MMP13 and increased the survival rates of MSCs. H_2_O_2_-induced (0.1 mM) changes in cytokines were attenuated in the extract and nanoparticles by pretreatment and cotreatment at two time points (*p* < 0.05). H_2_O_2_ increased cell apoptosis. In contrast, treatment with nanoparticle-loaded TS suppressed the percentage of apoptosis considerably (*p* < 0.05). Therefore, TS may be considered as a potential candidate for enhancing the effectiveness of MSC transplantation in cell therapy.

## 1. Introduction

*Thymus serpyllum* is traditionally used as a culinary herb. In folk medicine, it is used for the prevention or treatment of certain diseases [1]. The phenolic monoterpenes, thymol and carvacrol, are the major components of the plant. Some studies have indicated that this herb contains a polyphenol which is the main reason for its antioxidant effect when used as an aqueous tea [2]. For decades, naturally derived antioxidants have been gaining more attention in the natural medical domain. Among the antioxidants, polyphenols are widely used as a source of alternative and complimentary therapy for various diseases, including cancers. Polyphenols extracted from rice or isolated polyphenols manifest specific biological properties such as antimicrobial, antifungal, anti-inflammatory, and free radical scavenging activities in vitro and in vivo. Previous studies have shown that the water extract of the plant *Thymus serpyllum* has potential antioxidant and antihypersensitive effects in vitro. In traditional practice, plants rich in polyphenols have been consumed in the form of water extracts, especially as herbal teas. Despite the advantage of these plant-based polyphenols, some limitations do exist, like loss of its biological property due to poor storage conditions and the unpleasant taste of phenol. These limitations were resolved by encapsulating plant extracts into nanoparticles which would reduce the decomposition of the polyphenol and improve the slow release of polyphenols in the gastrointestinal system [3].

Encapsulated formulations of *T. serpyllum* have a potential to be used as additives to new functional food products. Upon intake of such products, it is possible to achieve a synergistic action of different polyphenolic compounds. Numerous studies have proved the pharmacological properties of polyphenols, including antioxidative, anti-inflammatory, and antimutagenic properties. Polyphenols are plant metabolites which act as powerful antioxidants, i.e., they neutralize the harmful effects of free radicals and thus provide support to the immune system. Among the more than 8000 phenolic compounds, each one is structurally very different, and the majority are presented in the form of phenolic acids, flavanols, flavonoids, and dihydrochalcones. The main feature of these compounds is the presence of one aryl ring attached with the hydroxyl group [4, 5].

Oxidative stress is one of the major factors underlying the pathogenesis of many diseases. Hence, the excessive production of free radicals could have a negative effect on the survival of transplanted stromal cells. Increased levels of reactive oxygen/nitrogen species (ROS/RNS) are associated with tissue injury and inflammation; they affect a number of cellular processes, including cell adhesion, migration, and proliferation; and they have been linked to cellular senescence in MSCs, potentially compromising their activities [6]. Though the use of stem cells as a therapeutic tool has shown great promise for treating various ailments such as cornea repair, blood vessel damage linked to heart attacks, or diseases such as critical limb ischemia, the efficacy of these treatments has not been established yet. Nevertheless, the major limitation seems to be the poor viability of the transplanted stromal cells in the injured site affecting its therapeutic efficacy [7]. Naturally occurring polyphenolic compounds (polyphenols), such as epigallocatechin-3-gallate (EGCG) and curcumin, block ROS/RNS and are potent inflammation-modulating agents [8].

A previous study has stated that chitosan/polyphenol systems could be a very promising functional food additive when used in combination with polymers with protective and mucoadhesive properties. In addition to the existing studies, incorporation of polyphenolic compounds in chitosan micro/nanoparticles has been achieved by spray drying or ionic gelation in the presence of polyphenolic compounds [9, 10], while other encapsulation technologies have not yet been explored enough. This study was designed to investigate the protective effect of *Thymus serpyllum* extract and nanoparticle-loaded plant extract on H_2_O_2_-induced damage of MSC.

## 2. Materials and Methods

### 2.1. Reagents

Analytical grade n-hexane and ascorbic acid were purchased from Sigma-Aldrich (Karlsruhe, Germany). All spectrophotometric data were collected using a Jasco V-530 UV-vis spectrophotometer (Jasco International Co. Ltd., Tokyo, Japan).

### 2.2. Preparation of Extract- and Nanoparticle-Loaded *Thymus serpyllum*

Dried plant material (*Thymus serpyllum*) was purchased during the flowering period (June-August) from a local market and was stored under cool and dark conditions. For the purpose of extract preparation, 60 g of dried plant material (flowers and leaves of *Thymus serpyllum*) were ground and weighed. The plant material was soaked in 800 ml of n-hexane for 72 hr at room temperature with occasional shaking. This was repeated five times and the mixture was subsequently filtered (Whatman Filter Paper No. 1, UK). The extract was concentrated to dryness using a rotary evaporator. The extract was stored at 4°C till further use. Chitosan beads were synthesized by applying the emulsion technique, with the addition of a crosslinking agent. For the preparation and encapsulation of *Thymus serpyllum* in chitosan nanoparticles, the protocol of Kata et al. [11] was followed. Briefly, chitosan microparticles (about 0.25 g) were immersed in 10 ml of thyme extract and the pH was adjusted to 3.5 with itaconic acid and left for 24 h in a mild orbital shaker. This will improve the capacity of the nanomaterials to absorb polyphenols from the aqueous extract. After that, the microparticles were filtered from the solution and dried in an oven at 37°C and subsequently in a vacuum until reaching constant weight; then, it was stored in a desiccator at room temperature.

### 2.3. Bone Marrow Stromal Cell Culture

Human bone marrow mesenchymal cells were isolated from bone marrow samples from donors at the University of Malaya Medical Center. The patient samples were collected based on written informed consent, and other approved guidelines were followed. 2.5 ml of bone marrow was diluted with 2 ml of phosphate-buffered saline (PBS; pH 7.2), and Ficoll-Paque Premium was used to layer the sample (GE Healthcare Life Sciences, Sweden). The sample was subjected to gradient centrifugation at 1800 rpm for 30 min (Eppendorf 5810R). The collected monolayer was washed twice with low-glucose Dulbecco's modified Eagle's medium (DMEM) (Invitrogen-Gibco, USA) containing 1% antibiotic/antimycotic (v/v; Invitrogen-Gibco). The isolated mononuclear cells were cultured in a low-glucose DMEM containing 10% fetal bovine serum with antibiotics and GlutaMAX (Invitrogen-Gibco). Cells were transferred into tissue culture flasks (Nunc™, USA) and cultured periodically every 3 days of media change.

Human bone marrow-derived mesenchymal stromal cells were isolated in accordance with the standard laboratory protocol. For the control group, cells were untreated. Another group of cells were treated with H_2_O_2_ (0.05 mM and 0.1 mM for a 2 hr incubation period). Following isolation, these cells were cultured in the DMEM medium (Invitrogen, Carlsbad, CA, USA) and supplemented with 10% stem cell-specified fetal bovine serum (FBS, Invitrogen), 100 U/ml penicillin (Sigma-Aldrich, USA), and 100 mg/ml streptomycin (Sigma-Aldrich). Cells were cultivated in tissue culture flasks at 37°C in a humidified atmosphere of 5% CO_2_. Upon achieving 80% confluence, cells were harvested with trypsin (Cell Applications, San Diego, CA, USA) and passaged. Cells used in this study were obtained from a control donor (28- to 40-year age group). Exclusion criteria include the following: emergency operation for infection (skin, soft tissue, or bone) and chronic osteoarthritis, history of osteosarcoma, medical history contraindicating bone marrow aspiration, history of prior or concurrent diagnosis of HIV, hepatitis-B, or hepatitis-C infection, and diagnosis of diabetes or autoimmune disease. Inclusion criteria include the following: patients under total knee replacement, ACL reconstruction, and fracture treatment and patients whose donors are able to understand and accept the aspiration procedure.

### 2.4. Cell Seeding

Human mesenchymal stromal cells were trypsinized and detached at passage 3 and seeded onto a 35 mm petri dish with a cell density of 30,000–40,000 cells. The medium was changed at specific time points (24, 48, and 72 hr) according to the respective treatment plan.

### 2.5. Gas Chromatography–Mass Spectroscopic (GC–MS) Analysis

An Agilent Technologies 7890A Network Gas Chromatographic (GC) system equipped with a TOF-MS (mass selective) detector and an Agilent Technologies 7693 series automatic liquid sampler were used for the analysis of the hexane extract. The injection volume was 1.0 *μ*l with split ratio of 1 : 10. An electron ionization system producing an ionization energy of 70 eV was used for GS-MS analysis of the sample. Initial temperature was set at 40°C (hold time of 5-minute interval). Furthermore, the settings of 4°C/min at 160°C and 5°C/min at 280°C with a 10-minute hold time were used. Gas (helium) was provided at a flow rate of 1.0 ml/min and at the mass scanning range (50–1000 m/z). The temperatures for the injector (250°C) and MS transfer line (300°C) were set.

#### 2.5.1. Free Radical Scavenging Activity

The free radical scavenging potential of the hexane extract was assessed by measuring its ability to scavenge 2,2′-diphenyl-1-picrylhydrazyl stable radicals (DPPH). The samples (2 to 10 mg/ml) were mixed with 1 ml of 90 *μ*mol/l DPPH solution and made up with 95% methanol to attain a final volume of 4 ml. Ascorbic acid was used as control. After an incubation period of 30 minutes at room temperature, the absorbance was recorded at 515 nm. Percent of radical scavenging concentration was calculated using the following formula:

(1) $$\begin{array}{c} \text{Radical scavenging}\left(\%\right)=100\times \left(\frac{A\text{ blank}-A\text{ sample}}{A\text{ blank}}\right), \end{array}$$

where (*A* blank) is the absorbance of the control (ascorbic acid)—containing all reagents except the test extract—and (*A* sample) is the absorbance of each tested extract. IC50 values representing the concentration of the extract that caused 50% scavenging were calculated from the plot of percentage scavenging against concentration.

### 2.6. Immunocytochemistry

Cells on each grid plate were fixed with 4% (w/v) paraformaldehyde (PFA, Sigma-Aldrich) in 1x phosphate-buffered saline for 20 min at room temperature. Cells were treated for 5 min and thrice washed with the PBS 0.1% (v/v) Triton X-100 (Sigma-Aldrich) in 1 × PBS. To block nonspecific binding, the cells were incubated with 2% (v/v) goat serum (Sigma-Aldrich) in 1 × PBS for 30 min at room temperature and washed thrice with the PBS. The cells were incubated with primary antibodies at 4°C for 3 h. The following primary antibodies (anti-FN antibody IST-9) were used for incubation (1 : 1000; Abcam, England): B-cell lymphoma 2 (BCL2), cytochrome c (Cyt-c), and matrix metalloproteinase 13 (MMP13). After the incubation, the cells were washed thrice with 1 × PBS for 5 min each. Polyclonal secondary antibody (chicken) was added to Anti-Mouse IgG FITC labelled (Abcam, England), and 1 × PBS was added for double staining. These cells were incubated for 1 h at room temperature and counterstained using Hoechst dye and kept for 15 minutes. The signals were observed under a microscope (Nikon C-HGFI, Japan), and the images were taken documented NIS elemental imaging software using the NIS*-*Elements Documentation software.

### 2.7. Cytokine Assay and Flow Cytometry

ProcartaPlex™ Multiplex Immunoassays for serum, plasma, and cell culture supernatants were used to examine the cytokine levels. The cells were collected by centrifugation, and the supernatant was aspirated. The cells were resuspended in 0.5–1.0 ml 1 × PBS, and formaldehyde was added to obtain a final concentration of 45%. This was fixed for 10 min at 37°C. The tubes were chilled on ice for 1 min. Cells were permeabilized by adding ice-cold methanol slowly to prechilled cells, while gently vortexing to a final concentration of 90% methanol. It was further incubated for 30 min on ice. Immunostaining was done with Section D, or cells were stored at −20°C in 90% methanol. 0.5–1 × 10^5^ cells were aliquoted into each assay tube, and 2–3 ml incubation buffer was added to each tube and washed by centrifugation. Cells were resuspended in 100 *μ*l of primary antibody (prepared in incubation buffer as directed) and incubated for 1 hour at room temperature. Washing by centrifugation in 2–3 ml of incubation buffer resuspended the cells in 0.5 ml 1 × PBS and this was analyzed on a flow cytometer. Cells were resuspended in 0.5 ml of DNA dye (propidium iodide) and incubated for at least 30 min at room temperature. Cells were analyzed in DNA staining solution on a flow cytometer.

### 2.8. Statistical Analysis

All the experiments were performed in triplicate and the data are presented as mean values ± standard deviation of triplicate findings. Statistical analysis of the data was performed using the SPSS Program, and a probability value of *p* ≤ 0.001 was considered to show a statistically significant difference.

## 3. Results

**Figure 1:**
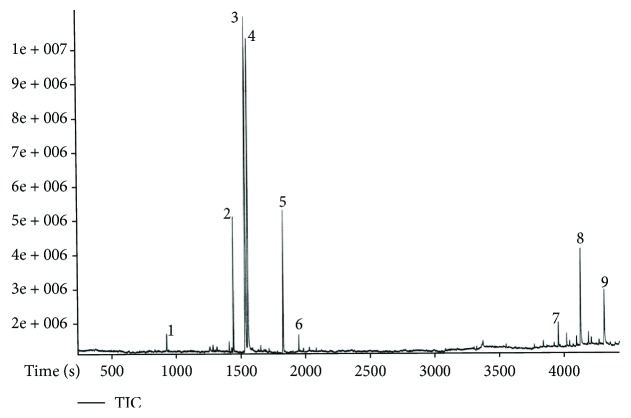
Gas chromatography–mass spectroscopy chromatogram of *Thymus serpyllum* hexane extract. Peak numbers indicate the type of active component present in the extract.

**Figure 2:**
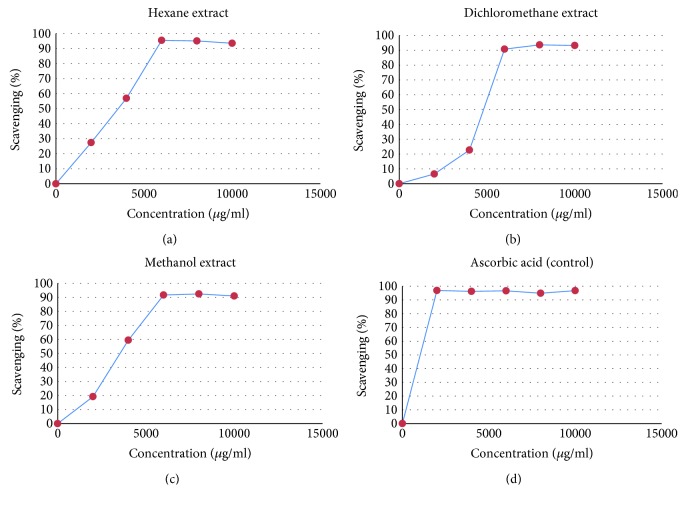
Free radical scavenging potential of (a) hexane extract, (b) dichloromethane extract, (c) methanol extract, and (d) ascorbic acid (control). The percentage of scavenging activity has been compared with ascorbic acid used as control.

The extract was subjected to GC-MS chromatography. The list of the chemical compositions is shown in Table 1, and their respective spectra are shown in Figure 1. In vitro free radical scavenging activity was examined using a different extra hexane extract, dichloromethane extract, and methanol extract, with ascorbic acid used as a positive control as shown in Figure 2(d). The concentration of 10,000 *μ*g/ml showed approximately 90% free radical scavenging activity, while 5000 *μ*g/ml of hexane extract (Figure 2(a)) showed approximately 40–50% free radical scavenging activity. While dichloromethane (Figure 2(b)) and methanol (Figure 2(c)) extract at 5000 *μ*g/ml showed around 20–25% free radical scavenging activity, increased concentrations showed around 80–90%, respectively.

**Figure 3:**
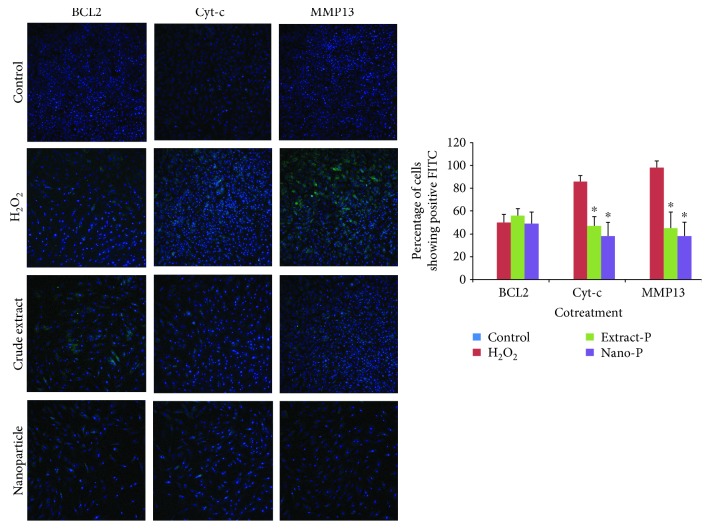
Expression of BCL2, cytochrome c (Cyt-c), and matrix metalloproteinase (MMP13) in control (untreated) and experimental groups (Extract-S and Nano-S represent cotreatment with hydrogen peroxide (H_2_O_2_–0.5 mM)).

**Figure 4:**
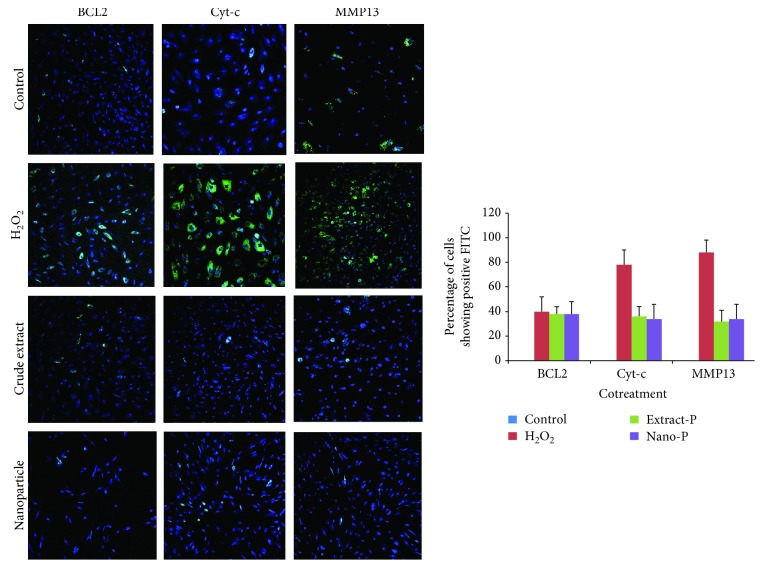
Expression of BCL2, cytochrome c (Cyt-c), and matrix metalloproteinase (MMP13) in control (untreated) and experimental groups (Extract-S and Nano-S represent cotreatment with hydrogen peroxide (H_2_O_2_—1 mM)).

**Figure 5:**
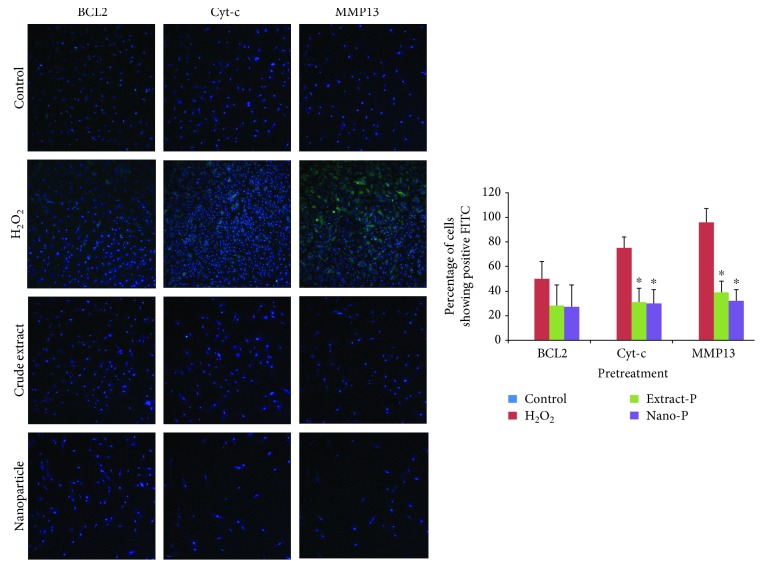
Expression of BCL2, cytochrome c (Cyt-c), and matrix metalloproteinase (MMP13) in control (untreated) and experimental groups (Extract-P and Nano-P represent pretreatment with hydrogen peroxide (H_2_O_2_–0.05 mM)).

**Figure 6:**
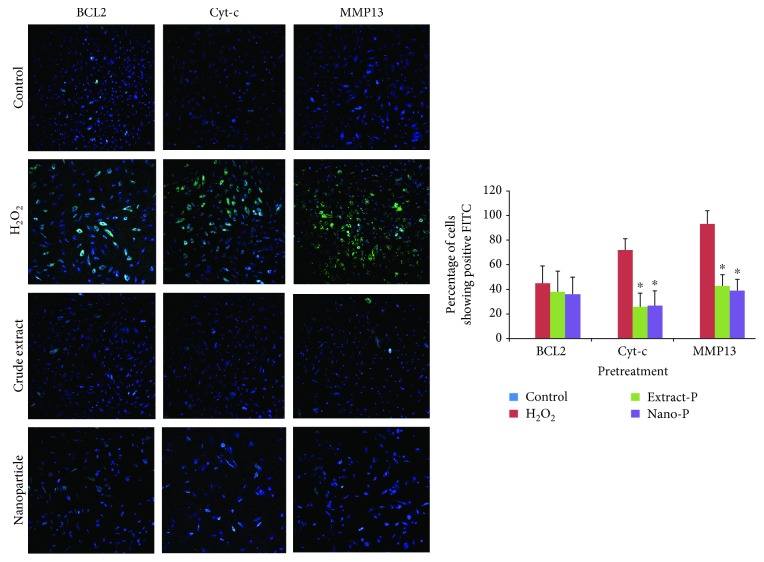
Expression of BCL2, cytochrome c (Cyt-c), and matrix metalloproteinase (MMP13) in control (untreated) and experimental groups (Extract-P and Nano-P represent pretreatment with hydrogen peroxide (H_2_O_2_—0.1 mM)).

Initially, bone marrow stromal cells were exposed to each of the two concentrations of hydrogen peroxide (0.05 mM and 0.1 mM, 2 h) at 37°C to test the potential of the extract and nanoparticle-loaded plant extract (Figures 3–6). Expression of BCL2, MMP13, and cytochrome c was examined. The control group showed no or very less percentage of cells positive for FITC BCL2 expression. The H_2_O_2_ treatment induced 40–50% of cells to be positive for FITC BCL2 expression, when compared with those of the extract treatment group. The cells treated with nanoparticle-loaded plant extract showed BCL2 expression, however it was statistically not significant. We also examined the expression of the other two important factors of apoptosis, cytochrome c and MMP13. The bone marrow stromal cells treated with H_2_O_2_ induced 72–86% cell expression of cytochrome c, while the cotreatment with extract and nanoparticles induced 47% and 38% cell expression, respectively, which was statistically (*p* < 0.05) less significant when compared to cells treated with H_2_O_2_ alone. The percentage of cells that appeared to be positive for FITC cells of the MMP13 antibody was around 88–98%, while for the cotreatment groups, either extract or nanoparticles, it was around 45% and 38%, respectively. These results indicated that the extract and nanoparticles cotreated against hydrogen peroxide were effective. When the concentration of hydrogen peroxide was increased and cotreated with the extract and nanoparticles, the expression of BCL2 was almost similar with the H_2_O_2_-treated group. However, the Cyto-c and MMP13 expression was considerably less when compared with the H_2_O_2_-only group. This is indicative that the extract and nanoparticles could be protective against apoptosis in cells. However, the nonexpressive level of BCL2 against apoptosis was not evident which needs further investigation.

Isolated human bone marrow stromal cells were pretreated with extract and nanoparticles and challenged against H_2_O_2._ There was no difference between the cells positive for FITC (BCL2) when treated with H_2_O_2_ only and pretreated with extract and nanoparticles. However, the extract pretreatment significantly reduced the expression of Cyt-c (26%–31%) and MMP13 (39%–43%), while the nanoparticle pretreatment showed the expression of Cyt-c (27%–30%) and MMP13 (32%–39%) when compared with the cells treated with H_2_O_2_.

**Figure 7:**
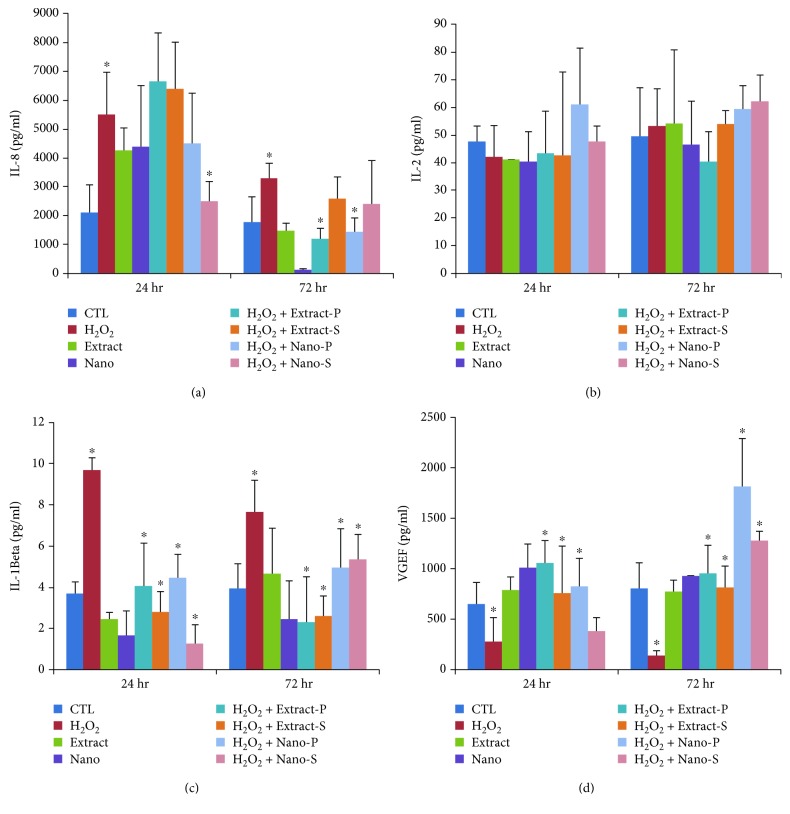
(a–d) Levels (pg/ml) of IL8, IL2, IL1beta, and VEGF in control (untreated) and experimental groups (S—cotreatment, P—pretreatment), as well nano- and extract-treated cells at two different time points, 24 hr and 72 hr.

Furthermore, the levels of cytokines were examined in bone marrow stromal cells treated with H_2_O_2_ at two time points, namely 24 h and 72 h. The cells treated with H_2_O_2_ induced the release of IL8 (Figure 7(a)) by approximately 2-fold when compared to the untreated control cells. Furthermore, the cells cotreated with nanoparticles inhibited the release when compared with those that were treated with H_2_O_2_ only. At 72 h, the release was considerably reduced (*p* < 0.05) in extract pretreatment and nanoparticle cotreatment. No significant changes were observed in the levels of IL2 (Figure 7(b)). IL*β*1 was significantly increased by 2-fold in the cells treated with H_2_O_2_ at 24 h and 72 h time points compared to the control (Figure 7(c)). It was also observed that the cotreatment and pretreatment with extract and nanoparticles significantly reduced the increase in IL2 by 2-fold (*p* < 0.05). Next, the vascular endothelial growth factor was significantly decreased by 2-fold when compared with the control. The levels were considerably reduced in cotreatment and pretreatment with extract and nanoparticles at 72 h (Figure 7(d)). Cells cotreated with nanoparticles did not prevent the decrease significantly at 24 h time, while the extract cotreatment, extract pretreatment, and nanoparticle pretreatment prevented the decrease.

**Figure 8:**
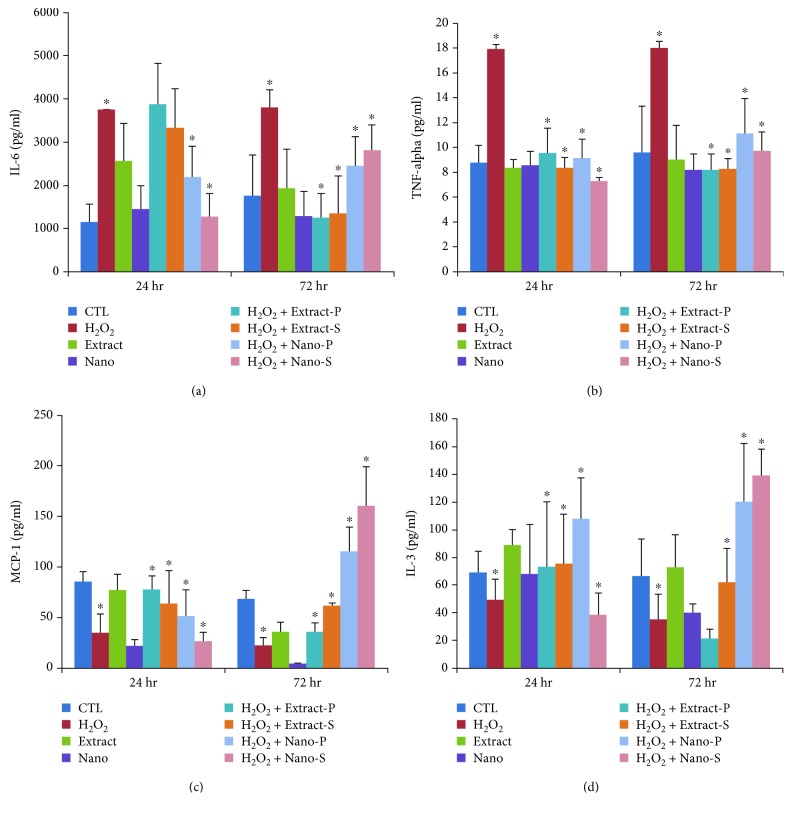
(a–d) Levels (pg/ml) of IL6, IL2, TNF-alpha, MCP1, and IL3 in control (untreated) and experimental groups (S—cotreatment, P—pretreatment), as well nano- and extract-treated cells at two different time points, 24 hr and 72 hr.

In addition, we examined the levels of IL6 (Figure 8(a)), which showed that the cells treated with H_2_O_2_ induced the release of IL6, a proinflammatory factor, by approximately 3-fold in comparison to the control. In response to this, the levels were reduced in nanoparticle cotreatment and pretreatment while the extract showed a protective role at 72 h (*p* < 0.05) when compared with the cells treated with H_2_O_2_ only.

The levels of TNF-*α* in the cells treated with H_2_O_2_ (Figure 8(b)) increased significantly when compared to control cells. In contrast, the levels did not increase significantly in the cells treated with extract and nanoparticles in both cotreatment and pretreatment. The levels of MCP in the cells treated with H_2_O_2_ considerably decreased (Figure 8(c)) when compared with the untreated control cells. These decreases were significantly prevented when the cell extract or nanoparticles were cotreated or pretreated. The levels of IL3 in the cells treated with H_2_O_2_ decreased when compared with the control cells. In contrast, extract and nanoparticle-loaded plant extract were not decreased (*p* < 0.05) compared with untreated cells at the 24 h and 72 h time points (Figure 8(d)).

**Figure 9:**
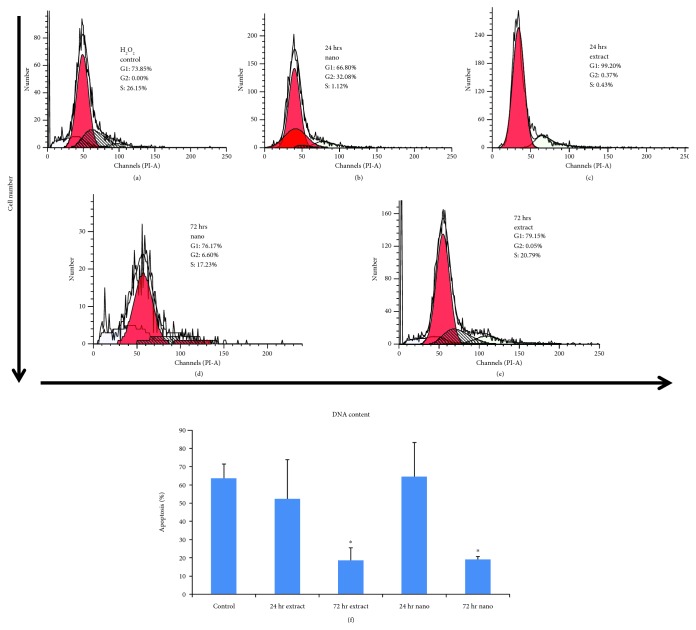
Flow cytometric analysis of H_2_O_2_ control and experimental groups such as extract- and nanoparticle-loaded *Thymus serpyllum* treated to cells at two different time points, 24 hr and 72 hr, as cotreatment against H_2_O_2_ (a–e). Relative percentage of apoptosis (f) in control and experimental groups such as extract- and nanoparticle-loaded *Thymus serpyllum* treated to cells at two different time points, 24 hr and 72 hr, as cotreatment against H_2_O_2_.

All the hMSCs were treated similarly with both nanoparticle and extract preparations, and the distribution of cells in different phases of the cell cycle was analyzed (Figure 9(a)–9(e)). Treatment with nanoparticle-loaded plant extract led to a marginal accumulation of cells in the G2/M phase following pretreatment time. However, after 72 h of similar treatment, the arrest of the cells in the G1 and S phases of the cell cycle was observed. In the case of cotreatment with extracted cells, both 24 h and 72 h had no effect on the G2/M phase of the cell cycle, except for a marginal increase in the S phase (in case of 72 h). Together, these results clearly show that the observed cell growth effects of both nanoparticle and extract preparations were associated with their cell cycle arrest activity. The percentage of apoptosis was also calculated from FACS data, as shown in Figure 9(f). The control- (H_2_O_2_) treated cells showed a high percentage of cells undergoing apoptosis, while nanoparticle-loaded plant extract-treated cells and plant extract-treated cells showed a low apoptosis percentage at 72 h; however, no effect was observed at 24 h.

## 4. Discussion

For decades, the use of polyphenols involves an aqueous extraction process of herbs and preparation of herbal tea [7]. During this preparation, the biological effects may be affected due to the presence of oxygen moisture and storage conditions. In addition, the consumption of polyphenols is limited due to their taste. Such limitations can be improved via encapsulation methods like converting liquid to solid forms for easier consumption. A further advantage of encapsulation is having a slow release formula. These methods have potential in new functional food products [12]. Previous studies on aqueous *Thymus serpyllum* L. extract have proved that it has a pronounced antioxidant and free radical scavenging activity in vitro and an antihypertensive effect. So, for different compounds like PLGA and PLA, hydrogen has been used for the encapsulation of active molecules [13]. Although chitosan/polyphenol systems could be very promising as a functional food additive, to our knowledge there has been only one report on the entrapment of thyme polyphenols into chitosan microbeads. In addition, in the existing studies, incorporation of polyphenolic compounds in chitosan micro/nanoparticles has been performed by spray drying or ionic gelation in the presence of polyphenolic compounds, while other encapsulation technologies have not yet been explored adequately enough. A previous study attempted to load polyphenolic compounds into chitosan microbeads that were obtained by an emulsion crosslinking technique (ready-made support) by the so-called postloading entrapment. They have examined the physiochemical properties of the bead, while the biological property or radical quenching property was not examined [14, 15].

Among different cell sources, mesenchymal stem cells show an extensive differentiation potential abundant in bone marrow tissue, adipose tissue, and several other tissues. The adipose or bone marrow stromal cells are a good source for potential applications due to their abundance, accessibility, and low immunogenicity [16]. Their survival and rate of differentiation at the injured site of transplantation is limited due to apoptosis in the stress environment. Therefore, the identification of a strategy for the protection of transplanted MSCs is key for the development of MSC-based therapy for regenerative therapy [17].

In the present study, the HPLC chromatogram detected polyphenols in the *Thymus serpyllum* extract. Despite the wide spectrum of attractive biological properties, the protective effects of *Thymus serpyllum* on hydrogen peroxide-induced changes in MSCs have yet to be fully elucidated. The BCL2 family of proteins is composed of the antiapoptotic proteins and proapoptotic proteins, of which their relative proportions control the fine balance between cell survival and cell death via the intrinsic apoptotic pathway [18]. The present study indicated that fluorescence microscopy could reverse the reduction of the antiapoptotic protein BCL2 following treatment with H_2_O_2_, but the level of reduction was not significant. In addition, cotreatment with extract and nanoparticle-loaded extract with H_2_O_2_ significantly reduced the Cyto-c and MMP13 levels compared with cells treated with H_2_O_2_ alone, showing the protective effects of the extract or nanoparticle of *Thymus serpyllum*. Reactive oxygen species (ROS) excessively produced by the respiratory chain can also cause progressive mitochondrial damage leading to apoptosis. All of the proposed cytochrome c releasing mechanisms, however, remain hypothetical, and fail to provide a physiological basis for the underlying naturally occurring apoptosis. In support of our data, the change in the expressions of *MMP13* was remarkably directly upregulated by the oxidant, and their activities were implicated in the invasive potential induced in cells [19]. The activation of *MMP* probably occurs by the reaction of ROS with thiol groups in the protease catalytic domain. In addition to their role as key regulators of *MMP* activation, ROS have been implicated in *MMP* gene expression [20]. Since treatment reduced their elevation in the MMP, it is an indication of its protective role on oxidative stress-mediated damage to the cells.

Interleukin (IL8) is a cytokine with potent chemotactic properties for neutrophils and T lymphocytes, and thus serves to amplify the inflammatory cascade. IL8 is produced by a wide variety of cell types including mononuclear cells, endothelial cells, and epithelial cells [21], and it has been implicated as an important mediator of neutrophil infiltration. However, some studies have shown no direct relation with apoptosis in other cell lines, and the increase in stromal cells upon H_2_O_2_ treatment was indicative of apoptosis in relation with the increase in TNF-alpha when compared to the control group left untreated. As shown in the previous study on other cell lines, we speculate that as a potent chemoattractant IL8 secretion stimulated by the Fas ligation might be associated with inflammation, and that it might participate in the pathophysiology of apoptosis. MSCs secrete cytokines either “spontaneously” or after induction by other cytokines, the most important being IL6, TNF*α*, and IL1*β* [22, 23], and it should be underlined that MSCs are not always immunosuppressive. It is assumed that their effects are determined by the local conditions of the microenvironment, and sometimes the proinflammatory IL6, TNF*α*, and IL1*β* cytokines may induce cell death upon H_2_O_2_ treatment, which could have been considerably prevented upon the treatment with *Thymus serpyllum*, indicative of protective property. However, the mechanism of action is undisclosed and further studies are warranted. The induction of other proinflammatory factors by IL6 has been regarded as part of an attempt to maintain homeostasis. Similarly, the coincidence of the induction of necrotic cell death signaling with the induction of proinflammatory signaling molecules, such as IL6, may function to alert the cells of the occurrence of necrotic cell death with consequent removal of the necrotic cells during the cell cycle. *Thymus serpyllum* treatment showed considerable protection in maintaining the homeostasis against hydrogen peroxide-induced changes in cytokine levels.

Previous studies have demonstrated that MSC secreted paracrine factors that are able to induce angiogenesis and affect cellular migration. Unique to our study, we quantified the secretion of VEGF and MCP1 by MSCs into the surrounding media, and we were able to detect the same factors in MSCs, while these factors declined after H_2_O_2_ treatment. A report has shown that [24] human embryonic stem cells released paracrine factors that reduced apoptosis in H9c2 cells, and it focused on TIMP-1 (tissue inhibitor of metalloproteinase) as an important molecule in this process. Other investigators have identified a number of factors secreted by cord blood-derived [25] and embryonic stem cell-derived [26] MSCs including VEGF and MCP1, but they did not determine their specific biological effects. The treatment with extract and nanoparticles showed a positive improvement in the levels of antiapoptotic factors. Interleukin (IL3), a cytokine secreted by activated T lymphocytes, is known to regulate hematopoiesis. Previously, reports have shown that IL3 prevents bone and cartilage damage in animal models of human rheumatoid arthritis and osteoarthritis [27, 28]. IL3 also promotes the differentiation of human MSCs into functional osteoblasts and increases their in vivo regenerative potential in immunocompromised mice. However, the role of IL3 in the migration and motility of MSCs is not yet known. In this study, we investigated the role of IL3 on human MSCs isolated from BM treated with H_2_O_2_ following pre- and cotreatment with extract or with nanoparticles of *Thymus serpyllum*. We found that IL3 significantly enhanced the migration and motility of MSCs, which can help the cells treated with the extract or nanoparticles.

## 5. Conclusion

Both *Thymus serpyllum* extract and nanoparticles showed considerable protection against hydrogen peroxide-induced mesenchymal stromal cell damage in vitro. Hence, this nanoparticle-loaded *Thymus serpyllum* can be a potential candidate to use as an adjuvant treatment with stem cell treatment.

## Figures and Tables

**Table 1:** List of chemical compounds identified in the hexane extract of *Thymus serpyllum* using GC-MS.

Compounds	Formula	Retention time (s)	Similarity	Weight	Area %
Benzene, 1-methyl-3-(1-methylethyl)-	C_10_H_14_	926.85	971	134	2.3176
Thymoquinone	C_10_H_12_O_2_	1444.55	950	164	8.8140
Thymol	C_10_H_14_O	1540.2	956	150	35.2661
Carvacrol	C_10_H_14_O	1559.65	954	150	30.9641
p-*tert*-Butyl catechol	C_10_H_14_O_2_	1830.75	788	166	9.8301
2(4H)-Benzofuranone, 5,6,7,7a-Tetrahydro-4,4,7a-trimethyl-	C_11_H_16_O_2_	1984.85	899	180	1.4065
2-Methyloctacosane	C_29_H_60_	3840.35	931	408	1.5172
Hexacosane	C_26_H_54_	4127.25	961	366	2.3650
Heptacosane	C_27_H_56_	4310.75	960	380	7.4996

## Data Availability

The data used to support the findings of this study are available from the corresponding author upon request. We assure data availability on reviewer's request.
